# How to make hand hygiene interventions more attractive to nurses: A discrete choice experiment

**DOI:** 10.1371/journal.pone.0202014

**Published:** 2018-08-09

**Authors:** Qian Zhao, Miles M. Yang, Yu-Ying Huang, Wenlin Chen

**Affiliations:** 1 School of Information Science and Engineering, Chengdu University, Chengdu, Sichuan, China; 2 Macquarie University, Sydney, Australia; 3 Healthcare Services Department, Commerce Development Research Institute, Taipei, Taiwan; 4 Business School, The University of New South Wales, Sydney, Australia; University of New South Wales, AUSTRALIA

## Abstract

**Background:**

Better understanding of the characteristics of interventions which are attractive to nurses is required in order to implement effective hand hygiene interventions.

**Methods:**

The intervention characteristics were derived from diffusion of innovation theory (DIT): relative advantage, compatibility, simplicity, trialability, and observability. To identify nurses’ preferences for the five characteristics, a discrete choice experiment (DCE) was conducted. Participants were nurses working at Taiwanese tertiary care hospitals selected through stratified sampling. In addition, the hand hygiene moment (before or after patient contact) was taken into consideration in the DCE to investigate whether nurses’ preferences for the intervention characteristics were the same at different hand hygiene moments.

**Results:**

This survey was conducted between 1 October and 31 December 2014. Among 200 nurses from three Taiwanese tertiary care hospitals, significant preferences for the five intervention characteristics were observed. That is, when an intervention makes the hand hygiene activity more convenient (p<0.001), when nurses participate in the design of the intervention (p<0.001), when an intervention is explained well to nurses before implementing it (p<0.001), when the evidence of hand hygiene is provided at a trial stage to show its effectiveness (p<0.001), and when nurses’ hand hygiene performance is observable to their peers (p<0.001), nurses are more willing to wash their hands with high compliance. In addition, nurses preferred for providing evidence at a trial stage most, and well explanation about the intervention to increase simplicity was least. The rankings of the preference for the five intervention characteristics were the same at different hand hygiene moments (p = 0.453)

**Conclusions:**

The findings suggest policy directions for decision makers aiming to improve overall hand hygiene compliance in healthcare facilities.

## 1. Introduction

Although the importance of hand hygiene in preventing health care-associated infections (HCAIs) is clear, it has been observed that nurses’ hand hygiene compliance is generally low [1–4]. To improve compliance, extensive research has been undertaken on hand hygiene interventions, e.g., through educational programs, providing reminders in wards, changing hand hygiene products, promoting role models, and providing incentives [1, 5–8]. Of the implemented interventions, some can promote compliance significantly, but others cannot. To make interventions more effective and pronounced, common characteristics of interventions that make individuals more willing to comply with guidelines need to be identified in order to facilitate policy responses.

The extant discussion on intervention characteristics has been guided by diffusion of innovation theory (DIT). DIT, popularized by Roger [9, 10], considers how, why, and at what rate individuals adopt any innovation. DIT has been selected to identify characteristics of an attractive intervention because the purpose of an innovation is similar to that of an intervention, i.e., changing individuals’ behavior to achieve a certain goal. Rogers’ theory suggests that there are five characteristics of innovation which have influence on the adoption of innovation by individuals: relative advantage (to what extent an innovation is perceived as better than the idea it supersedes), compatibility (to what extent an innovation is perceived as consistent with individuals’ past experiences and existing values), simplicity (to what extent an innovation is perceived as difficult to understand), trialability (to what extent an innovation may be trialed), and observability (to what extent the results of an innovation are visible to others).

DIT provides characteristics which could affect the success of an intervention. However, the number of studies in the area is relatively small, and there is little study specifically discussing the five characteristics in the context of hand hygiene intervention [11–13]. In addition, the extant discussion of these characteristics is qualitative [13]. Without quantifying the impact of each characteristic, it is not known to what degree the characteristics of an intervention affect nurses’ willingness to improve their hand hygiene compliance.

A discrete choice experiment (DCE), as a stated preference method, was chosen in order to conduct the quantitative study. This is not only because DCE can systematically investigate nurses’ preferences for intervention characteristics, but also because DCE can study the influence of the characteristics before an intervention is actually implemented. This can be done by asking respondents to answer questions in hypothetical scenarios, rather than real situations.

DCEs have been widely used in the field of health care since the early 1990s to understand patients’ preferences for various health services [14, 15] or to evaluate health care workers’ (HCWs’) preferences about human resources policies [16, 17]. However, to our knowledge there is no published example using DCE to assess potential characteristics that may impact the effectiveness of interventions. Our study is the first DCE examining nurses’ preferences for hand hygiene intervention characteristics.

The purpose of this study is to use a quantitative approach to explore the characteristics of an attractive hand hygiene intervention. The insight into nurses’ preferences can help decision makers design and implement more effective and efficient interventions to improve overall hand hygiene compliance in a healthcare facility.

## 2. Methods

### 2.1 Ethics statement

The protocol and informed consent forms for this study were approved by Institutional Review Board in Commerce Development Research Institute, Taipei, Taiwan (Approval No. 101014). Participation was voluntary and all participants signed an informed consent form before any study procedure.

### 2.2 Development of attributes and levels

DCEs involve the careful design of choice sets in which two or more hypothetical alternatives are offered to respondents. They are asked to evaluate the options and choose the most preferred alternative in each choice set. Each hypothetical alternative is described by a package of attributes of a situation, such as factors that could affect nurses’ willingness to improve their hand hygiene compliance.

Table 1 summarizes the six attributes explored in this study. The first five were derived from DIT. To make the description of these attributes more appropriate in the context of hand hygiene intervention, we consulted with three experts and hosted two focus groups with nurses. Through the discussion with experts and focus groups, it was suggested to us that hand hygiene moment could have some influence on nurses’ hand hygiene compliance and their preferences to the intervention characteristics. Therefore, the hand hygiene moment was included in this study as the sixth attribute.

**Table 1:** Attributes and levels.

Attributes	Levels (Name)	Coding
ADVANTAGE	An intervention **can** make hand hygiene activity become significantly convenient (ADVANTAGE_yes)	1
	An intervention **cannot** make hand hygiene activity become significantly convenient (ADVANTAGE_no)*	-1
COMPATIBILITY	Nurses **are** provided with an opportunity to participate in the design of an intervention, and therefore, their hand hygiene behavior become more compatible with past experience (COMPATIBILITY_yes)	1
	Nurses **are not** provided with an opportunity to participate in the design of an intervention, and therefore, their hand hygiene behavior cannot become compatible with past experience (COMPATIBILITY_no)*	-1
SIMPLICITY	An intervention **is** explained well to nurses before implementing, and therefore the level of simplicity of the intervention for understanding it is increased (SIMPLICITY_high)	1
	An intervention **is not** explained well to nurses before implementing, and therefore the level of simplicity of the intervention for understanding it is not increased (SIMPLICITY_low)*	-1
TRIALABILITY	Evidence **is** provided at a trial stage to show the effectiveness of hand hygiene in reducing the prevalence level of HCAIs (TRIALABILITY_yes)	1
	Evidence **is not** provided at a trial stage to show the effectiveness of hand hygiene in reducing the prevalence level of HCAIs (TRIALABILITY_no)*	-1
OBSERVABILITY	Nurses’ hand hygiene performance is reported **publicly,** which is observable to the other peer nurses (OBSERVABILITY_yes)	1
	Nurses’ hand hygiene performance is reported **privately,** which is not observable to the other peer nurses (OBSERVABILITY_no)*	-1
MOMENT	**After** patient contact (MOMENT_after)	1
	**Before** patient contact (MOMENT_before)*	-1

*Reference level

The description of attributes was originally written in English. Then this was translated into traditional Chinese by two bilingual people and translated back into English by two different bilingual people, as recommended by Brislin [18] and Triandis [19].

### 2.3 Experimental design

As shown in Table 1, there were six attributes in the DCE, and each attribute had two levels. Therefore 64 (= 2^6^) different scenarios could be defined in a full factorial design, resulting in 2016 (= (64 × 63)/2) combinations of pairwise choices. Since the number of choice sets was too large to be manageable for the respondents, a subset of the scenarios was selected by SAS to obtain as much information as possible.

**Fig 1:**
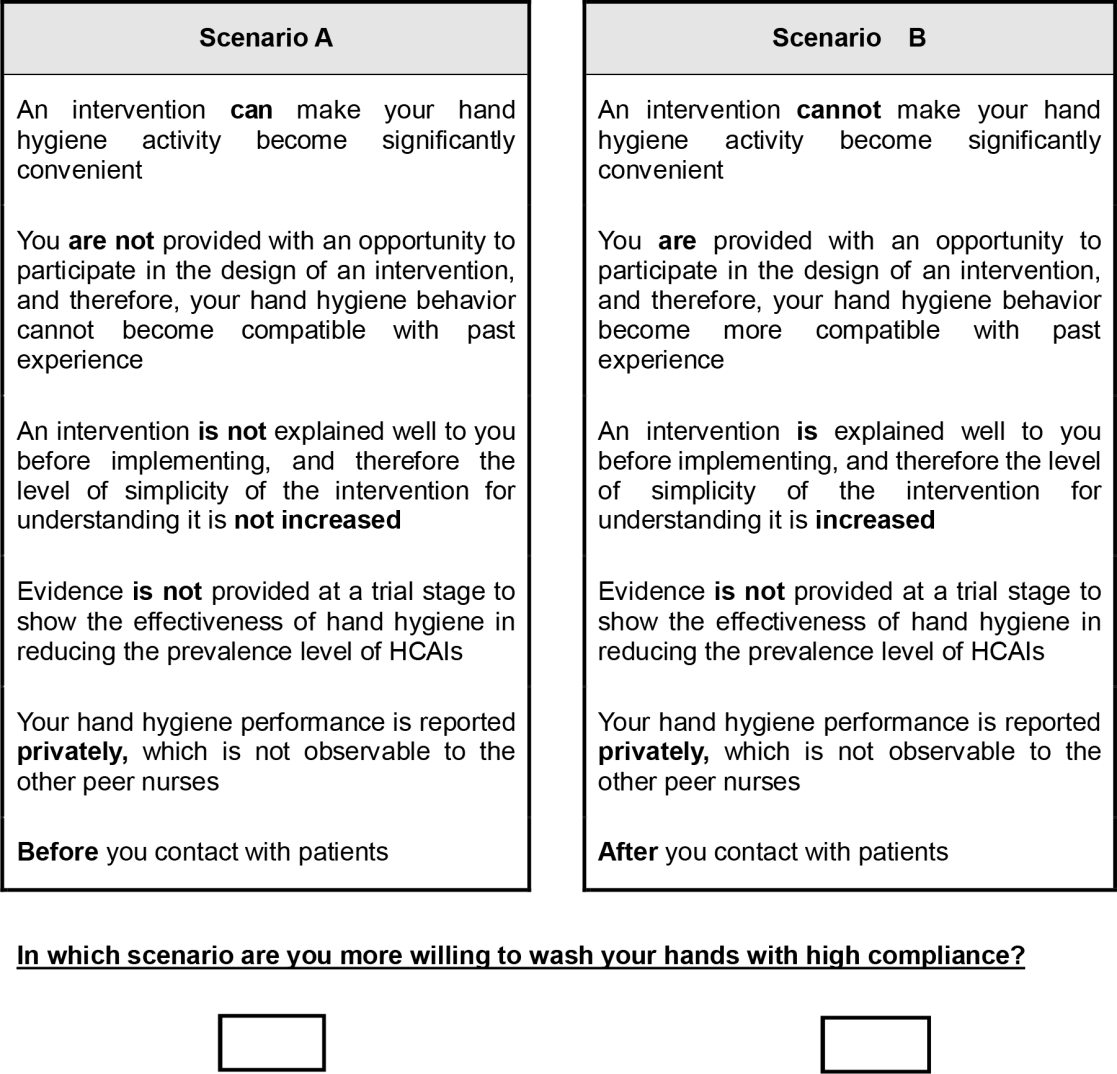
Sample choice set.

As a result, 32 choice sets were generated and each set included two hypothetic scenarios. In each set, nurses were asked to imagine that they were in the given scenarios, and to choose one of the scenarios in each choice set under which they were more willing to wash the hands with high compliance. Four versions of questionnaire were produced by SAS and each version had eight rather than 32 choice sets (S1 Text). This is because nurses were very busy. They were reluctant to systematically evaluate 32 choice sets at one time. An example of a DCE choice set is provided in Fig 1.

### 2.4 Recruitment of participants

Participants were nurses involved in daily patient-care activities. They were recruited from three Taiwanese tertiary care hospitals. Stratified sampling was used to randomly select the eligible participants in each hospital, generating a list of chosen nurses. A study investigator gave the list to the head of nurses in each hospital, and asked them to invite the nurses on the given list to complete the questionnaires.

In sample size, studies have shown that 20–30 respondents per version of questionnaire can provide precise parameter estimates [20]. Since we had four versions of the questionnaire, the appropriate sample size was 80–120. Considering the response rate, 220 questionnaires in total were distributed.

### 2.5 Data collection

The data were collected between 1 October and 31 December 2014. The choice sets were administered in an anonymous questionnaire. An example was given at the beginning of the survey to the participants on how to answer the choice questions and help them quickly understand the choice experiments. In addition, participants also needed to complete questions about their socio-demographic characteristics.

### 2.6 Data analysis

Econometric models of DCE are derived from random utility theory (RUT) [21, 22]. Based on the framework of RUT, a conditional logistic regression model was built to estimate the mean change in utility placed by nurses on attribute levels compared to the reference level (see S2 Text for more information about the econometric model used in this study). The estimations were presented by marginal utility values. A positive (negative) marginal utility value implies that an attribute level is preferred more (less) than the reference level. P-value was used to indicate whether the preference for an attribute level is significantly different from that for the reference level.

In addition, predicted probability analysis [23] was conducted to evaluate the relative importance for the five intervention characteristics. This analysis follows five steps: (1) define each attribute to its base level; (2) evaluate the probability in the base case; (3) systematically vary each attribute over its levels; (4) evaluate the probability in the new case; and (5) compare the probability in the base case and the new case to calculate percentage change in probability. In our study, the base attribute levels were the reference levels defined in Table 1.

## 3. Results

### 3.1 Participants

A total of 218 participants returned the questionnaire; 18 were excluded in this study because they did not finish answering all questions in the questionnaire (either the DCE part or socio-demographic part) (S1 Dataset). The final sample size was 200, exceeding the usual rules of thumb for DCE sample size estimation. In brief, most participants were female (N = 192 [96%]). Over half (N = 109 [54.5%]) were between 31 and 40 years old; about one third were no more than 30 years old (N = 73 [36.5%]). Most had more than 15 years working experience (N = 85 [42.5%]). Details of sociodemographic characteristics of participants are listed in S1 Table.

### 3.2 Estimation results

Table 2 presents the marginal utility values for the five intervention characteristics: relative advantage (Coeff. 0.1414; p<0.001), compatibility (Coeff. 0.1885; p<0.001), simplicity (Coeff. 0.1086; p<0.001), trialability (Coeff. 0.1987; p<0.001), and observability (Coeff. 0.1803; p<0.001). All five values are positive and statistically significant, which implies that an intervention with the five characteristics would encourage nurses to improve their hand hygiene compliance considerably. However, unlike the five characteristics, the sixth attribute in the DCE, hand hygiene moment, had little influence on nurses’ handwashing practice (Coeff. -0.0481; p = 0.453). This means that there is no difference in nurses’ willingness to comply with the guidelines before and after patient contact.

**Table 2:** Estimated marginal utility values for the DCE.

**Variables**	Marginal utility values*	**St. Err.**	**P-value**
ADVANTAGE_yes [no]	0.1414***	0.0283	<0.001
COMPATIBILITY_yes [no]	0.1885***	0.0274	<0.001
SIMPLICITY_high [low]	0.1086***	0.0274	<0.001
TRIALABILITY_yes [no]	0.1987***	0.0274	<0.001
OBSERVABILITY_yes [no]	0.1803***	0.0286	<0.001
MOMENT_after [before]	-0.0481	0.0641	0.453

*** Significant at 1%

** Significant at 5%

* Significant at 10%

### 3.3 Relative importance of attributes

**Fig 2:**
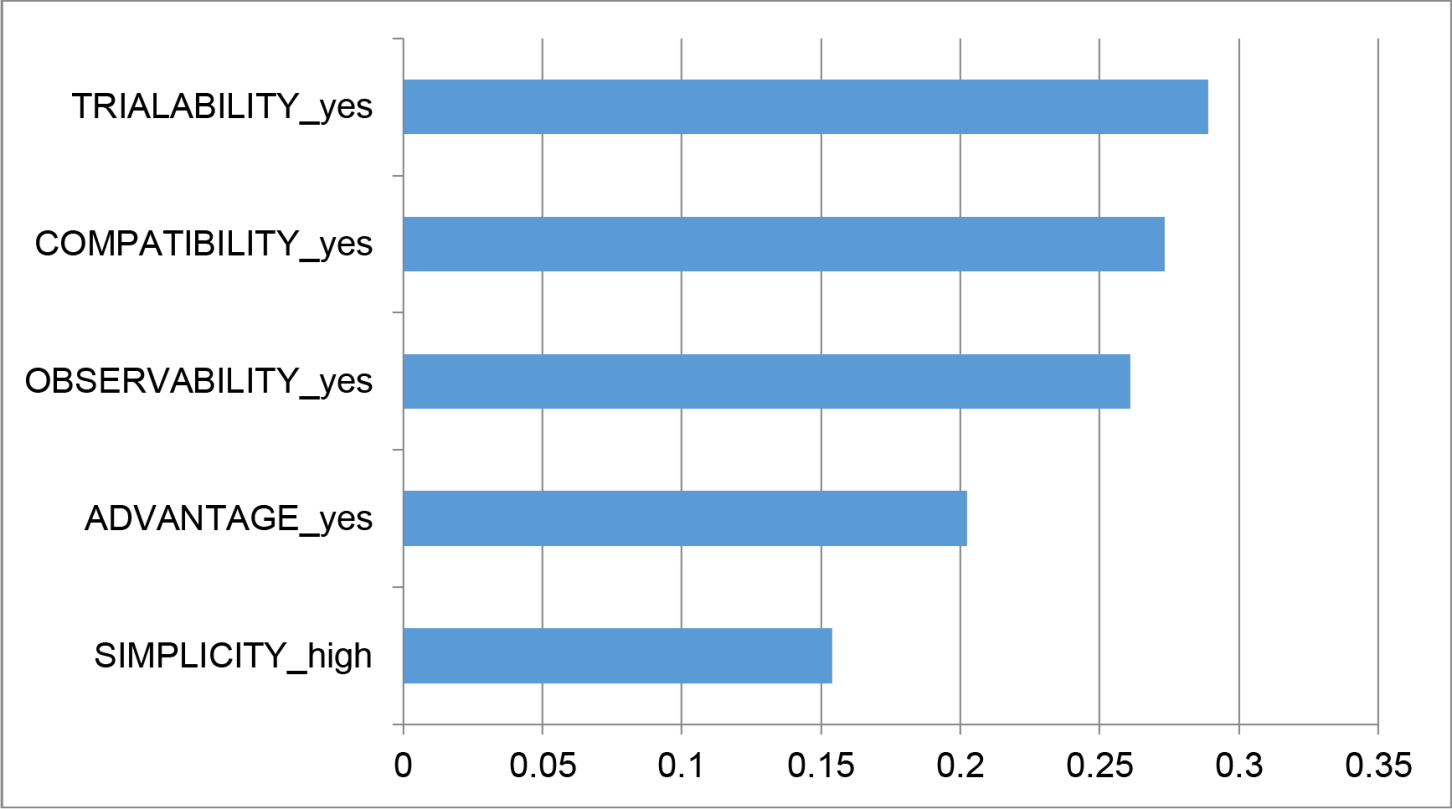
Relative importance of intervention characteristics.

To identify the relative importance of the five intervention characteristics, predicted probability analysis was conducted. In this analysis, the base case was defined that the intervention did not involve any characteristics. Without any attractive intervention characteristics, about 32% nurses were willing to wash their hands with high compliance. When varying intervention characteristics from the base level to the other, the ranking of importance of characteristics is shown in Fig 2. The most important characteristic was to provide evidence to nurses at trial stage to show the effectiveness of hand hygiene in infection control, resulting in a percentage change in probability by 28.89%. Participating in the design of hand hygiene intervention had the second greatest impact on the predicted probability, changing probability by 27.32%. Well explanation about the intervention to increase the simplicity for nurses’ understanding was the least important characteristic, only changing the probability by 15.4%.

In Fig 2, the hand hygiene moment was set to before patient contact (MOMENT = -1). In S1 Fig, the moment was set to after patient contact (MOMENT = 1). Comparison of comparing Fig 2 and the figure in S1 Fig shows that the rankings of the preferred intervention characteristics are the exactly same for the two different hand hygiene moments.

## 4. Discussion

### 4.1 Summary of findings

This study is the first not only using DIT in the context of hand hygiene intervention to provide insight into preferred intervention characteristics for promoting nurses’ hand hygiene behavior, but also presenting evidence to quantify the relative importance of these characteristics to nurses’ hand hygiene decisions. We found that five intervention characteristics, relative advantage (p<0.001), compatibility (p<0.001), simplicity (p<0.001), trialability (p<0.001), and observability (p<0.001), were very attractive to nurses to wash their hands with high compliance. Of the five characteristics, nurses preferred trialability most and simplicity least. We also found that the ranking of nurses’ preferences for the intervention characteristics before patient contact were the same as after patient contact.

### 4.2 Interpretation of findings

Nurses have the strongest preference for an intervention that provides solid evidence about the effectiveness of hand hygiene in reducing the prevalence of HCAIs at a trial stage. This is because presenting concrete evidence at a trial stage could reinforce the importance and advantages of hand hygiene, which would boost nurses’ confidence and therefore result in more positive attitudes towards handwashing practices [24]. The extant studies report that HCWs with more positive attitudes were much more likely to wash their hands according to the hand hygiene guidelines [25, 26].

Nurses will be more willing to improve their handwashing compliance if an intervention is compatible with their hand hygiene habits, past experiences and potential needs for hand hygiene. It is suggested that integrating nurses in the design of an intervention could make the intervention more compatible [27]. A popular way of integration is to invite HCWs to participate in an open discussion or a focus group to address their motivations for and barriers to hand hygiene [28–31].

Nurses tended to comply with hand hygiene guidelines if their handwashing performance could be observed by their peers. This finding is in line with previous studies [30, 32–37]. One explanation is that delivering the hand hygiene performance to patient-care groups rather than individuals, or posting in communal places, makes nurses no longer ‘hide in the crowd’, and therefore, they may feel the pressure from their peers if the probability of their peers’ hands being contaminated increases, which will drive them to improve their hand hygiene compliance [24, 38–41].

Nurses also showed a preference for an intervention which can make hand hygiene activity more convenient. With convenient access to supplies at the point of care, they do not need to go and wash their hands at another location and then return to continue to work, which could facilitate nurses following hand hygiene guidelines [30, 39].

The effectiveness of an intervention was also influenced by the characteristic of simplicity. Evidence shows that difficulty in understanding an intervention is one major reason of non-effective hand hygiene intervention [29, 42, 43]. This is because, with complex and unclear explanations about the intervention, nurses do not understand the rationale for performing all hand hygiene practices. As a result, they are less motivated by the intervention to wash their hands.

### 4.3 Strengths and limitations of methods

The strength of this study is identification of nurses’ preferences for intervention characteristics using a DCE survey, which allows us to investigate multiple factors influencing nurses’ hand hygiene compliance and rank the importance of these factors. Based on an appropriate sample size and a high response rate from three Taiwanese hospitals, our study produced meaningful and robust estimates. There were some limitations to our study. Our study represents the stated choice of nurses working in Taiwanese hospitals, and it could not capture incentives about the other types of HCWs in the same area or in other areas. However, with the same framework as the one in this study, future study could compare preferences for intervention characteristics across different types of HCWs, HCWs with different socio-demographic factors, or HCWs in other countries. Although attributes and levels in the DCE were developed from the literature, expert opinions and focus group interviews, each attribute about intervention characteristics had two levels, with and without that characteristic, only providing general insights about whether the existence of these characteristics could attract nurses to improve their hand hygiene compliance. In future, researchers could develop more levels for each intervention characteristic according to their own settings to make this study more specific. It is not clear that whether participants are going to actually make the same decisions in real life as they stated in the DCE. This is because, in addition to the attributes discussed in the experiment, there are several factors in real life that can affect nurses’ hand hygiene decisions. The actual impact of intervention characteristics can be captured fully through longitudinal studies. However, valuable preliminary information can be provided by DCEs to improve the understanding of individuals’ behaviors and incentives for behavior change.

## 5. Conclusions

The most attractive intervention characteristic for nurses to comply with the guidelines is to provide solid evidence of handwashing practices at a trial stage to show its effectiveness of infection control. In addition, an intervention that makes hand hygiene activity become more convenient, provides opportunities to nurses to participate in the design of intervention, is well-explained to nurses before implementing, and allows nurses’ hand hygiene performance to be observable to their peers, is also helpful to promote their handwashing compliance.

## Supporting information

S1 Text Questionnaires. (DOCX)

S2 Text Details of data analysis. (DOCX)

S1 Dataset DCE data from participants in this study. (DTA)

S1 Table Sociodemographic characteristics of participants. (DOCX)

S1 Fig Relative importance of intervention characteristics after patient contact. (TIF)
