# Supplementary material for: How to make hand hygiene interventions more attractive to nurses: A discrete choice experiment
Source: PLoS One. 2018 Aug 9;13(8):e0202014. doi: 10.1371/journal.pone.0202014 (PMC6084975; doi:10.1371/journal.pone.0202014)
Supplement: S1 Text — (DOCX) [file pone.0202014.s001.docx]

S1 Text. Questionnaires

**VERSION 1**

**INTRODUCTION TO THE SURVEY**

The purpose of this survey is to help us understand your hand hygiene behavior in daily patient care activities. This survey has two parts.

PART I asks about your basic information. There are seven questions in this part.

In PART II, you will be given two **hypothetic** scenarios, A and B. Each scenario is defined by six factors. You need to imagine that you are in the given scenarios, and then choose one of the scenarios in each question under which you are more willing to comply with guidelines and wash your hands with high compliance. There are eight questions in this part.

**When you make the choice in the hypothetic question, we ask that you take it seriously, consider it carefully, and respond it as if you were actually facing the situation described in the question. Your answer will contribute to identifying the factors and improving the overall hand hygiene compliance in the hospital.**

In total, there will be 15 questions. The survey may take up to 15 minutes to complete. You can decide whether to participate in this study. Your decision will not have any influence on your performance evaluation. Any information obtained in this survey will remain confidential.

**PART I: YOUR BASIC INFORMATION**

**1.1** Your name: ______________________

**1.2** You are a

①Nurse ②Medical doctor ③Nursing student ④Medical student ⑤Other

**1.3** Your age is

①<20 ②20-25 ③26-30 ④31-40 ⑤>40

**1.4** How many years have you worked as a healthcare worker?

①<1 ②1-3 ③4-8 ④9-14 ⑤15-20 ⑥>20

**1.6** Your education level is

①Bachelor ②Master ③Doctor ④Other

**1.6** Your gender is

①Male ②Female

**1.7** You are working in **(*This question is only for a nurse or a nursing student*)**

①Intensive care unit ②General ward ③Other

**PART II**

**PLEASE READ**

- *PART II has 8 choice questions. In each question, you are given two* ***hypothetic*** *scenarios, A and B. Please* ***imagine*** *you were in the given scenarios, and* ***choose one of the scenarios*** *in* ***each question*** *under which you are* ***more willing*** *to comply with guidelines and wash your hands with high compliance.*
- *Please place a “√” in the chosen box.*
- *An example about a choice question is given next.*

**EXAMPLE OF A CHOICE QUESTION**

| **Scenario A** | |  | **Scenario B** | |
| --- | --- | --- | --- | --- |
| An intervention can make your hand hygiene activity become significantly convenient | |  | An intervention cannot make your hand hygiene activity become significantly convenient | |
| You are provided with an opportunity to participate in the design of an intervention, and therefore, your hand hygiene behavior become more compatible with past experience | |  | You are not provided with an opportunity to participate in the design of an intervention, and therefore, your hand hygiene behavior cannot become compatible with past experience | |
| An intervention is not explained well to you before implementing, and therefore the level of simplicity of the intervention for understanding it is not increased | |  | An intervention is explained well to you before implementing, and therefore the level of simplicity of the intervention for understanding it is increased | |
| Evidence is not provided at a trial stage to show the effectiveness of hand hygiene in reducing the prevalence level of HCAIs | |  | Evidence is not provided at a trial stage to show the effectiveness of hand hygiene in reducing the prevalence level of HCAIs | |
| Your hand hygiene performance is reported privately, which is not observable to the other peer nurses | |  | Your hand hygiene performance is reported privately, which is not observable to the other peer nurses | |
| Before you contact with patients | |  | After you contact with patients | |
|  | |  |  | |
| **In which scenario are you more willing to wash your hands with high compliance?** | | | | |
| *√* |  | | |  |

*In this example, Scenario A is chosen. This means that the respondent is more willing to wash the hands with high compliance in Scenario A.*

**2.1**

| **Scenario A** | |  | **Scenario B** | |
| --- | --- | --- | --- | --- |
| An intervention cannot make your hand hygiene activity become significantly convenient | |  | An intervention can make your hand hygiene activity become significantly convenient | |
| You are not provided with an opportunity to participate in the design of an intervention, and therefore, your hand hygiene behavior cannot become compatible with past experience | |  | You are provided with an opportunity to participate in the design of an intervention, and therefore, your hand hygiene behavior become more compatible with past experience | |
| An intervention is explained well to you before implementing, and therefore the level of simplicity of the intervention for understanding it is increased | |  | An intervention is not explained well to you before implementing, and therefore the level of simplicity of the intervention for understanding it is not increased | |
| Evidence is not provided at a trial stage to show the effectiveness of hand hygiene in reducing the prevalence level of HCAIs | |  | Evidence is provided at a trial stage to show the effectiveness of hand hygiene in reducing the prevalence level of HCAIs | |
| Your hand hygiene performance is reported privately, which is not observable to the other peer nurses | |  | Your hand hygiene performance is reported publicly, which is observable to the other peer nurses | |
| Before you contact with patients | |  | Before you contact with patients | |
|  | |  |  | |
| **In which scenario are you more willing to wash your hands with high compliance?** | | | | |
|  |  | | |  |

**2.2**

| **Scenario A** | |  | **Scenario B** | |
| --- | --- | --- | --- | --- |
| An intervention can make your hand hygiene activity become significantly convenient | |  | An intervention cannot make your hand hygiene activity become significantly convenient | |
| You are provided with an opportunity to participate in the design of an intervention, and therefore, your hand hygiene behavior become more compatible with past experience | |  | You are not provided with an opportunity to participate in the design of an intervention, and therefore, your hand hygiene behavior cannot become compatible with past experience | |
| An intervention is not explained well to you before implementing, and therefore the level of simplicity of the intervention for understanding it is not increased | |  | An intervention is explained well to you before implementing, and therefore the level of simplicity of the intervention for understanding it is increased | |
| Evidence is provided at a trial stage to show the effectiveness of hand hygiene in reducing the prevalence level of HCAIs | |  | Evidence is not provided at a trial stage to show the effectiveness of hand hygiene in reducing the prevalence level of HCAIs | |
| Your hand hygiene performance is reported publicly, which is observable to the other peer nurses | |  | Your hand hygiene performance is reported privately, which is not observable to the other peer nurses | |
| After you contact with patients | |  | After you contact with patients | |
|  | |  |  | |
| **In which scenario are you more willing to wash your hands with high compliance?** | | | | |
|  |  | | |  |

**2.3**

| **Scenario A** | |  | **Scenario B** | |
| --- | --- | --- | --- | --- |
| An intervention can make your hand hygiene activity become significantly convenient | |  | An intervention cannot make your hand hygiene activity become significantly convenient | |
| You are not provided with an opportunity to participate in the design of an intervention, and therefore, your hand hygiene behavior cannot become compatible with past experience | |  | You are provided with an opportunity to participate in the design of an intervention, and therefore, your hand hygiene behavior become more compatible with past experience | |
| An intervention is not explained well to you before implementing, and therefore the level of simplicity of the intervention for understanding it is not increased | |  | An intervention is explained well to you before implementing, and therefore the level of simplicity of the intervention for understanding it is increased | |
| Evidence is provided at a trial stage to show the effectiveness of hand hygiene in reducing the prevalence level of HCAIs | |  | Evidence is not provided at a trial stage to show the effectiveness of hand hygiene in reducing the prevalence level of HCAIs | |
| Your hand hygiene performance is reported publicly, which is observable to the other peer nurses | |  | Your hand hygiene performance is reported privately, which is not observable to the other peer nurses | |
| Before you contact with patients | |  | Before you contact with patients | |
|  | |  |  | |
| **In which scenario are you more willing to wash your hands with high compliance?** | | | | |
|  |  | | |  |

**2.4**

| **Scenario A** | |  | **Scenario B** | |
| --- | --- | --- | --- | --- |
| An intervention cannot make your hand hygiene activity become significantly convenient | |  | An intervention can make your hand hygiene activity become significantly convenient | |
| You are not provided with an opportunity to participate in the design of an intervention, and therefore, your hand hygiene behavior cannot become compatible with past experience | |  | You are provided with an opportunity to participate in the design of an intervention, and therefore, your hand hygiene behavior become more compatible with past experience | |
| An intervention is explained well to you before implementing, and therefore the level of simplicity of the intervention for understanding it is increased | |  | An intervention is not explained well to you before implementing, and therefore the level of simplicity of the intervention for understanding it is not increased | |
| Evidence is not provided at a trial stage to show the effectiveness of hand hygiene in reducing the prevalence level of HCAIs | |  | Evidence is provided at a trial stage to show the effectiveness of hand hygiene in reducing the prevalence level of HCAIs | |
| Your hand hygiene performance is reported privately, which is not observable to the other peer nurses | |  | Your hand hygiene performance is reported publicly, which is observable to the other peer nurses | |
| After you contact with patients | |  | After you contact with patients | |
|  | |  |  | |
| **In which scenario are you more willing to wash your hands with high compliance?** | | | | |
|  |  | | |  |

**2.5**

| **Scenario A** | |  | **Scenario B** | |
| --- | --- | --- | --- | --- |
| An intervention can make your hand hygiene activity become significantly convenient | |  | An intervention cannot make your hand hygiene activity become significantly convenient | |
| You are provided with an opportunity to participate in the design of an intervention, and therefore, your hand hygiene behavior become more compatible with past experience | |  | You are not provided with an opportunity to participate in the design of an intervention, and therefore, your hand hygiene behavior cannot become compatible with past experience | |
| An intervention is explained well to you before implementing, and therefore the level of simplicity of the intervention for understanding it is increased | |  | An intervention is not explained well to you before implementing, and therefore the level of simplicity of the intervention for understanding it is not increased | |
| Evidence is provided at a trial stage to show the effectiveness of hand hygiene in reducing the prevalence level of HCAIs | |  | Evidence is not provided at a trial stage to show the effectiveness of hand hygiene in reducing the prevalence level of HCAIs | |
| Your hand hygiene performance is reported privately, which is not observable to the other peer nurses | |  | Your hand hygiene performance is reported publicly, which is observable to the other peer nurses | |
| Before you contact with patients | |  | Before you contact with patients | |
|  | |  |  | |
| **In which scenario are you more willing to wash your hands with high compliance?** | | | | |
|  |  | | |  |

**2.6**

| **Scenario A** | |  | **Scenario B** | |
| --- | --- | --- | --- | --- |
| An intervention cannot make your hand hygiene activity become significantly convenient | |  | An intervention can make your hand hygiene activity become significantly convenient | |
| You are provided with an opportunity to participate in the design of an intervention, and therefore, your hand hygiene behavior become more compatible with past experience | |  | You are not provided with an opportunity to participate in the design of an intervention, and therefore, your hand hygiene behavior cannot become compatible with past experience | |
| An intervention is explained well to you before implementing, and therefore the level of simplicity of the intervention for understanding it is increased | |  | An intervention is not explained well to you before implementing, and therefore the level of simplicity of the intervention for understanding it is not increased | |
| Evidence is not provided at a trial stage to show the effectiveness of hand hygiene in reducing the prevalence level of HCAIs | |  | Evidence is provided at a trial stage to show the effectiveness of hand hygiene in reducing the prevalence level of HCAIs | |
| Your hand hygiene performance is reported publicly, which is observable to the other peer nurses | |  | Your hand hygiene performance is reported privately, which is not observable to the other peer nurses | |
| Before you contact with patients | |  | Before you contact with patients | |
|  | |  |  | |
| **In which scenario are you more willing to wash your hands with high compliance?** | | | | |
|  |  | | |  |

**2.7**

| **Scenario A** | |  | **Scenario B** | |
| --- | --- | --- | --- | --- |
| An intervention cannot make your hand hygiene activity become significantly convenient | |  | An intervention can make your hand hygiene activity become significantly convenient | |
| You are not provided with an opportunity to participate in the design of an intervention, and therefore, your hand hygiene behavior cannot become compatible with past experience | |  | You are provided with an opportunity to participate in the design of an intervention, and therefore, your hand hygiene behavior become more compatible with past experience | |
| An intervention is not explained well to you before implementing, and therefore the level of simplicity of the intervention for understanding it is not increased | |  | An intervention is explained well to you before implementing, and therefore the level of simplicity of the intervention for understanding it is increased | |
| Evidence is provided at a trial stage to show the effectiveness of hand hygiene in reducing the prevalence level of HCAIs | |  | Evidence is not provided at a trial stage to show the effectiveness of hand hygiene in reducing the prevalence level of HCAIs | |
| Your hand hygiene performance is reported publicly, which is observable to the other peer nurses | |  | Your hand hygiene performance is reported privately, which is not observable to the other peer nurses | |
| After you contact with patients | |  | After you contact with patients | |
|  | |  |  | |
| **In which scenario are you more willing to wash your hands with high compliance?** | | | | |
|  |  | | |  |

**2.8**

| **Scenario A** | |  | **Scenario B** | |
| --- | --- | --- | --- | --- |
| An intervention can make your hand hygiene activity become significantly convenient | |  | An intervention cannot make your hand hygiene activity become significantly convenient | |
| You are not provided with an opportunity to participate in the design of an intervention, and therefore, your hand hygiene behavior cannot become compatible with past experience | |  | You are provided with an opportunity to participate in the design of an intervention, and therefore, your hand hygiene behavior become more compatible with past experience | |
| An intervention is explained well to you before implementing, and therefore the level of simplicity of the intervention for understanding it is increased | |  | An intervention is not explained well to you before implementing, and therefore the level of simplicity of the intervention for understanding it is not increased | |
| Evidence is provided at a trial stage to show the effectiveness of hand hygiene in reducing the prevalence level of HCAIs | |  | Evidence is not provided at a trial stage to show the effectiveness of hand hygiene in reducing the prevalence level of HCAIs | |
| Your hand hygiene performance is reported privately, which is not observable to the other peer nurses | |  | Your hand hygiene performance is reported publicly, which is observable to the other peer nurses | |
| After you contact with patients | |  | After you contact with patients | |
|  | |  |  | |
| **In which scenario are you more willing to wash your hands with high compliance?** | | | | |
|  |  | | |  |

**THE END**

**版本１**

**調查問卷說明**

您好！

非常感謝您能抽出寶貴的時間參加問卷調查。我們希望通過這份問卷能夠更好的了解您在工作中的洗手行為。該問卷包含兩大部分。

第一部分為基本資料，共7題。

第二部分中的每個問題將會給出兩個**假設**場景，場景A 和B。每一個假設場景由六個因素組成。您需要想像您正處於所給出的假設場景中，通過比較場景A和B來選擇出一個讓您更加願意保持高洗手遵從性的場景，共8題。

**請注意：第二部分的問題均為假設性問題。當您在回答這些問題時，我們希望您能夠認真並且仔細考慮每一個問題. 即使所给定的場景可能不是您真實面臨的場景。您的回答將會對我們找出能夠有效提高醫護人員洗手遵從性的干預措施有很大的幫助。**

該問卷總共有15個問題，填答時間大約會花費您15分鐘。您也可以自由決定退出，您的決定不會對您的權益產生任何影響。問卷中所涉及的個人信息及隱私將會絕對保密。

**第一部分: 基本資料**

1.1 請寫下您的姓名

1.2 您是

①護士 ②醫生 ③實習護士 ④實習醫生 ⑤其他(請說明)

1. 3 您的年齡

①<20 ②20-25 ③26-30 ④31-40 ⑤>40

1.4 您從事醫護人員的工作已經有多少年了？

①<1 ② 1-3 ③4-8 ④9-14 ⑤15-20 ⑥>20

1.5 您的受教育程度

①大專 ②碩士 ③博士 ④其他 (請說明)

1.6 您的性别

①男 ②女

1.7 您的工作地點***（請注意:該問題僅限於護士和實習護士作答）***

①加護病房 ②普通病房 ③其他(請說明)

**第二部分**

**請仔細閱讀**

- 第二部分共有8個問題。每個問題將會給出兩個**假設**場景，A和B。請**想像**您正處在假設場景中，并請**選擇出一個**讓您**比較願意**保持高洗手遵從性的場景
- 請在所選場景對應的框中打“√”
- 在作答前，請仔細閱讀以下範例

| **範例** |  | |  | | |
| --- | --- | --- | --- | --- | --- |
| **場景 A** |  | | **場景 B** | | |
| 洗手幹預措施能夠在很大程度上使您的洗手活動變得方便 |  | | 洗手幹預措施並不能夠在很大程度上使您的洗手活動變得方便 | | |
| 您有機會參與到洗手幹預措施的設計當中，因此幹預措施中所提倡的洗手行為與您之前的洗手行為具有一致性 |  | | 您沒有機會參與到洗手幹預措施的設計當中，因此幹預措施中提倡的洗手行為與您之前的洗手行為沒有一致性 | | |
| 在實行幹預措施前，醫院沒有對該措施進行詳細的介紹和說明，因此幹預措施並沒有變得簡單易懂 |  | | 在實行幹預措施前，醫院對該措施進行了詳細的介紹和說明，因此幹預措施變得簡單易懂 | | |
| 在幹預措施的試運行階段，醫院沒有提供證據表明洗手活動能夠有效地減少院內感染 |  | | 在幹預措施的試運行階段，醫院沒有提供證據表明洗手活動能夠有效地減少院內感染 | | |
| 醫院沒有在全院範圍內公佈洗手遵從性情況，因此您的同事並不瞭解您的洗手遵從性 |  | | 醫院沒有在全院範圍內公佈洗手遵從性情況，因此您的同事並不瞭解您的洗手遵從性 | | |
| 接觸病人之前 |  | | 接觸病人之後 | | |
| **在哪個場景下您比較願意保持高的洗手遵從性？** | | | | | |
| *√* | |  |  | | |
|  |  |  |  |  |  |

***在此範例中，當作答者仔細閱讀並且比較了場景A和場景B的不同之處後，選擇了情景A，亦即，相對於情景B，該作答者比較願意在情景A中保持高的洗手遵從性。***

| **場景 A** |  | **場景 B** | | |
| --- | --- | --- | --- | --- |
| 洗手幹預措施並不能夠在很大程度上使您的洗手活動變得方便 |  | 洗手幹預措施能夠在很大程度上使您的洗手活動變得方便 | | |
| 您沒有機會參與到洗手幹預措施的設計當中，因此幹預措施中提倡的洗手行為與您之前的洗手行為沒有一致性 |  | 您有機會參與到洗手幹預措施的設計當中，因此幹預措施中所提倡的洗手行為與您之前的洗手行為具有一致性 | | |
| 在實行幹預措施前，醫院對該措施進行了詳細的介紹和說明，因此幹預措施變得簡單易懂 |  | 在實行幹預措施前，醫院沒有對該措施進行詳細的介紹和說明，因此幹預措施並沒有變得簡單易懂 | | |
| 在幹預措施的試運行階段，醫院沒有提供證據表明洗手活動能夠有效地減少院內感染 |  | 在幹預措施的試運行階段，醫院提供了證據來表明洗手活動能夠有效地減少院內感染 | | |
| 醫院沒有在全院範圍內公佈洗手遵從性情況，因此您的同事並不瞭解您的洗手遵從性 |  | 醫院在全院範圍內公佈洗手遵從性情況，因此您的同事也瞭解您的洗手遵從性 | | |
| 接觸病人之前 |  | 接觸病人之前 | | |
| **在哪個場景下您比較願意保持高的洗手遵從性？** | | | | |
|  |  |  | | |
|  |  |  |  |  |

**2.1**

**2.2**

| **場景 A** |  | **場景 B** | | |
| --- | --- | --- | --- | --- |
| 洗手幹預措施能夠在很大程度上使您的洗手活動變得方便 |  | 洗手幹預措施並不能夠在很大程度上使您的洗手活動變得方便 | | |
| 您有機會參與到洗手幹預措施的設計當中，因此幹預措施中所提倡的洗手行為與您之前的洗手行為具有一致性 |  | 您沒有機會參與到洗手幹預措施的設計當中，因此幹預措施中提倡的洗手行為與您之前的洗手行為沒有一致性 | | |
| 在實行幹預措施前，醫院沒有對該措施進行詳細的介紹和說明，因此幹預措施並沒有變得簡單易懂 |  | 在實行幹預措施前，醫院對該措施進行了詳細的介紹和說明，因此幹預措施變得簡單易懂 | | |
| 在幹預措施的試運行階段，醫院提供了證據來表明洗手活動能夠有效地減少院內感染 |  | 在幹預措施的試運行階段，醫院沒有提供證據表明洗手活動能夠有效地減少院內感染 | | |
| 醫院在全院範圍內公佈洗手遵從性情況，因此您的同事也瞭解您的洗手遵從性 |  | 醫院沒有在全院範圍內公佈洗手遵從性情況，因此您的同事並不瞭解您的洗手遵從性 | | |
| 接觸病人之後 |  | 接觸病人之後 | | |
| **在哪個場景下您比較願意保持高的洗手遵從性？** | | | | |
|  |  |  | | |
|  |  |  |  |  |

**2.3**

| **場景 A** |  | **場景 B** | | |
| --- | --- | --- | --- | --- |
| 洗手幹預措施能夠在很大程度上使您的洗手活動變得方便 |  | 洗手幹預措施並不能夠在很大程度上使您的洗手活動變得方便 | | |
| 您沒有機會參與到洗手幹預措施的設計當中，因此幹預措施中提倡的洗手行為與您之前的洗手行為沒有一致性 |  | 您有機會參與到洗手幹預措施的設計當中，因此幹預措施中所提倡的洗手行為與您之前的洗手行為具有一致性 | | |
| 在實行幹預措施前，醫院沒有對該措施進行詳細的介紹和說明，因此幹預措施並沒有變得簡單易懂 |  | 在實行幹預措施前，醫院對該措施進行了詳細的介紹和說明，因此幹預措施變得簡單易懂 | | |
| 在幹預措施的試運行階段，醫院提供了證據來表明洗手活動能夠有效地減少院內感染 |  | 在幹預措施的試運行階段，醫院沒有提供證據表明洗手活動能夠有效地減少院內感染 | | |
| 醫院在全院範圍內公佈洗手遵從性情況，因此您的同事也瞭解您的洗手遵從性 |  | 醫院沒有在全院範圍內公佈洗手遵從性情況，因此您的同事並不瞭解您的洗手遵從性 | | |
| 接觸病人之前 |  | 接觸病人之前 | | |
| **在哪個場景下您比較願意保持高的洗手遵從性？** | | | | |
|  |  |  | | |
|  |  |  |  |  |

**2.4**

| **場景 A** |  | **場景 B** | | |
| --- | --- | --- | --- | --- |
| 洗手幹預措施並不能夠在很大程度上使您的洗手活動變得方便 |  | 洗手幹預措施能夠在很大程度上使您的洗手活動變得方便 | | |
| 您沒有機會參與到洗手幹預措施的設計當中，因此幹預措施中提倡的洗手行為與您之前的洗手行為沒有一致性 |  | 您有機會參與到洗手幹預措施的設計當中，因此幹預措施中所提倡的洗手行為與您之前的洗手行為具有一致性 | | |
| 在實行幹預措施前，醫院對該措施進行了詳細的介紹和說明，因此幹預措施變得簡單易懂 |  | 在實行幹預措施前，醫院沒有對該措施進行詳細的介紹和說明，因此幹預措施並沒有變得簡單易懂 | | |
| 在幹預措施的試運行階段，醫院沒有提供證據表明洗手活動能夠有效地減少院內感染 |  | 在幹預措施的試運行階段，醫院提供了證據來表明洗手活動能夠有效地減少院內感染 | | |
| 醫院沒有在全院範圍內公佈洗手遵從性情況，因此您的同事並不瞭解您的洗手遵從性 |  | 醫院在全院範圍內公佈洗手遵從性情況，因此您的同事也瞭解您的洗手遵從性 | | |
| 接觸病人之後 |  | 接觸病人之後 | | |
| **在哪個場景下您比較願意保持高的洗手遵從性？** | | | | |
|  |  |  | | |
|  |  |  |  |  |

**2.5**

| **場景 A** |  | **場景 B** | | |
| --- | --- | --- | --- | --- |
| 洗手幹預措施能夠在很大程度上使您的洗手活動變得方便 |  | 洗手幹預措施並不能夠在很大程度上使您的洗手活動變得方便 | | |
| 您有機會參與到洗手幹預措施的設計當中，因此幹預措施中所提倡的洗手行為與您之前的洗手行為具有一致性 |  | 您沒有機會參與到洗手幹預措施的設計當中，因此幹預措施中提倡的洗手行為與您之前的洗手行為沒有一致性 | | |
| 在實行幹預措施前，醫院對該措施進行了詳細的介紹和說明，因此幹預措施變得簡單易懂 |  | 在實行幹預措施前，醫院沒有對該措施進行詳細的介紹和說明，因此幹預措施並沒有變得簡單易懂 | | |
| 在幹預措施的試運行階段，醫院提供了證據來表明洗手活動能夠有效地減少院內感染 |  | 在幹預措施的試運行階段，醫院沒有提供證據表明洗手活動能夠有效地減少院內感染 | | |
| 醫院沒有在全院範圍內公佈洗手遵從性情況，因此您的同事並不瞭解您的洗手遵從性 |  | 醫院在全院範圍內公佈洗手遵從性情況，因此您的同事也瞭解您的洗手遵從性 | | |
| 接觸病人之前 |  | 接觸病人之前 | | |
| **在哪個場景下您比較願意保持高的洗手遵從性？** | | | | |
|  |  |  | | |
|  |  |  |  |  |

**2.6**

| **場景 A** |  | **場景 B** | | |
| --- | --- | --- | --- | --- |
| 洗手幹預措施並不能夠在很大程度上使您的洗手活動變得方便 |  | 洗手幹預措施能夠在很大程度上使您的洗手活動變得方便 | | |
| 您有機會參與到洗手幹預措施的設計當中，因此幹預措施中所提倡的洗手行為與您之前的洗手行為具有一致性 |  | 您沒有機會參與到洗手幹預措施的設計當中，因此幹預措施中提倡的洗手行為與您之前的洗手行為沒有一致性 | | |
| 在實行幹預措施前，醫院對該措施進行了詳細的介紹和說明，因此幹預措施變得簡單易懂 |  | 在實行幹預措施前，醫院沒有對該措施進行詳細的介紹和說明，因此幹預措施並沒有變得簡單易懂 | | |
| 在幹預措施的試運行階段，醫院沒有提供證據表明洗手活動能夠有效地減少院內感染 |  | 在幹預措施的試運行階段，醫院提供了證據來表明洗手活動能夠有效地減少院內感染 | | |
| 醫院在全院範圍內公佈洗手遵從性情況，因此您的同事也瞭解您的洗手遵從性 |  | 醫院沒有在全院範圍內公佈洗手遵從性情況，因此您的同事並不瞭解您的洗手遵從性 | | |
| 接觸病人之前 |  | 接觸病人之前 | | |
| **在哪個場景下您比較願意保持高的洗手遵從性？** | | | | |
|  |  |  | | |
|  |  |  |  |  |

**2.7**

| **場景 A** |  | **場景 B** | | |
| --- | --- | --- | --- | --- |
| 洗手幹預措施並不能夠在很大程度上使您的洗手活動變得方便 |  | 洗手幹預措施能夠在很大程度上使您的洗手活動變得方便 | | |
| 您沒有機會參與到洗手幹預措施的設計當中，因此幹預措施中提倡的洗手行為與您之前的洗手行為沒有一致性 |  | 您有機會參與到洗手幹預措施的設計當中，因此幹預措施中所提倡的洗手行為與您之前的洗手行為具有一致性 | | |
| 在實行幹預措施前，醫院沒有對該措施進行詳細的介紹和說明，因此幹預措施並沒有變得簡單易懂 |  | 在實行幹預措施前，醫院對該措施進行了詳細的介紹和說明，因此幹預措施變得簡單易懂 | | |
| 在幹預措施的試運行階段，醫院提供了證據來表明洗手活動能夠有效地減少院內感染 |  | 在幹預措施的試運行階段，醫院沒有提供證據表明洗手活動能夠有效地減少院內感染 | | |
| 醫院在全院範圍內公佈洗手遵從性情況，因此您的同事也瞭解您的洗手遵從性 |  | 醫院沒有在全院範圍內公佈洗手遵從性情況，因此您的同事並不瞭解您的洗手遵從性 | | |
| 接觸病人之後 |  | 接觸病人之後 | | |
| **在哪個場景下您比較願意保持高的洗手遵從性？** | | | | |
|  |  |  | | |
|  |  |  |  |  |

**2.8**

| **場景 A** |  | **場景 B** | | |
| --- | --- | --- | --- | --- |
| 洗手幹預措施能夠在很大程度上使您的洗手活動變得方便 |  | 洗手幹預措施並不能夠在很大程度上使您的洗手活動變得方便 | | |
| 您沒有機會參與到洗手幹預措施的設計當中，因此幹預措施中提倡的洗手行為與您之前的洗手行為沒有一致性 |  | 您有機會參與到洗手幹預措施的設計當中，因此幹預措施中所提倡的洗手行為與您之前的洗手行為具有一致性 | | |
| 在實行幹預措施前，醫院對該措施進行了詳細的介紹和說明，因此幹預措施變得簡單易懂 |  | 在實行幹預措施前，醫院沒有對該措施進行詳細的介紹和說明，因此幹預措施並沒有變得簡單易懂 | | |
| 在幹預措施的試運行階段，醫院提供了證據來表明洗手活動能夠有效地減少院內感染 |  | 在幹預措施的試運行階段，醫院沒有提供證據表明洗手活動能夠有效地減少院內感染 | | |
| 醫院沒有在全院範圍內公佈洗手遵從性情況，因此您的同事並不瞭解您的洗手遵從性 |  | 醫院在全院範圍內公佈洗手遵從性情況，因此您的同事也瞭解您的洗手遵從性 | | |
| 接觸病人之後 |  | 接觸病人之後 | | |
| **在哪個場景下您比較願意保持高的洗手遵從性？** | | | | |
|  |  |  | | |
|  |  |  |  |  |

**非常感謝您完成本次問卷調查！**
