# Supplementary material for: How to make hand hygiene interventions more attractive to nurses: A discrete choice experiment
Source: PLoS One. 2018 Aug 9;13(8):e0202014. doi: 10.1371/journal.pone.0202014 (PMC6084975; doi:10.1371/journal.pone.0202014)
Supplement: S2 Text — (DOCX) [file pone.0202014.s002.docx]

S2 Text. Details of data analysis

Econometric models of DCE are derived from random utility theory (RUT), which assumes that an individual’s utility for a particular choice is unobservable and it consists of explainable and random parts. The choice’s explainable part can be broken down into a set of attributes, which allows the significance of the attributes to be tested by choosing preferred alternatives. The choice’s random part is the unobserved influence, the unobserved preference, or the measurement error from the perspective of economics. This can be written in the following equation $U_{\mathrm{ijs}}=V_{\mathrm{ijs}}+\varepsilon_{\mathrm{ijs}}$, where $U_{\mathrm{ijs}}$ is an overall utility for individual $i$ facing alternative $j$ in choice set $s$, $V_{\mathrm{ijs}}$ is the explainable part of utility, and $\varepsilon_{\mathrm{ijs}}$ is the random component.

In this study, nurses had two choices: whether or not to wash the hands with high compliance. The choice rule is as follows. The probability for nurse i to choose an alternative, say ‘yes’, is equal to the probability that the utility of alternative ‘yes’ is greater than (or equal to) the utility of alternative ‘no’. That is, $P_{yes}=Pr\left( U_{yes}\geq U_{no} \right)$. This is equivalent to

$$P_{yes}=Pr\left( V_{yes}+\varepsilon_{yes}\geq V_{no}+\varepsilon_{no} \right)=Pr(\varepsilon_{yes}-\varepsilon_{no}\geq V_{no}-V_{yes})$$

DCE data were analyzed by using a conditional logit model, which is able to estimate the mean change in utility placed by respondents assigned to attribute levels compared to the reference level. The model fit to the utility function was:

$V_{\mathrm{ijs}}=\beta_{1}ADVANTAGE+\beta_{2}COMPATIBILITY+\beta_{3}SIMPLICITY+\beta_{4}trialability+\beta_{5}observability+\beta_{6}Moment.$

In the model, $\beta_{1}-\beta_{6}$ are coefficients describing individuals’ preference for each attribute level on average. A positive (negative) and significant coefficient indicates that an attribute level is preferred more (less) than the reference level.
