# Supplementary material for: How to make hand hygiene interventions more attractive to nurses: A discrete choice experiment
Source: PLoS One. 2018 Aug 9;13(8):e0202014. doi: 10.1371/journal.pone.0202014 (PMC6084975; doi:10.1371/journal.pone.0202014)
Supplement: S1 Table — (DOCX) [file pone.0202014.s004.docx]

S1 Table. Sociodemographic characteristics of participants

| Characteristics | N (%) |
| --- | --- |
| Sex |  |
| Male | 8 (4) |
| Female | 192 (96) |
| Age, years |  |
| <20 | 6 (3) |
| 20-25 | 24 (12) |
| 26-30 | 43 (21.5) |
| 31-40 | 109 (54.5) |
| 40+ | 18 (9) |
| Working experience, years |  |
| <1 | 20 (10) |
| 1-3 | 27 (13.5) |
| 4-8 | 10 (5) |
| 9-14 | 58 (29) |
| 15-20 | 77 (38.5) |
| 20+ | 8 (4) |
