# Supplementary figures and images for: How to make hand hygiene interventions more attractive to nurses: A discrete choice experiment
Source: PLoS One. 2018 Aug 9;13(8):e0202014. doi: 10.1371/journal.pone.0202014 (PMC6084975; doi:10.1371/journal.pone.0202014)

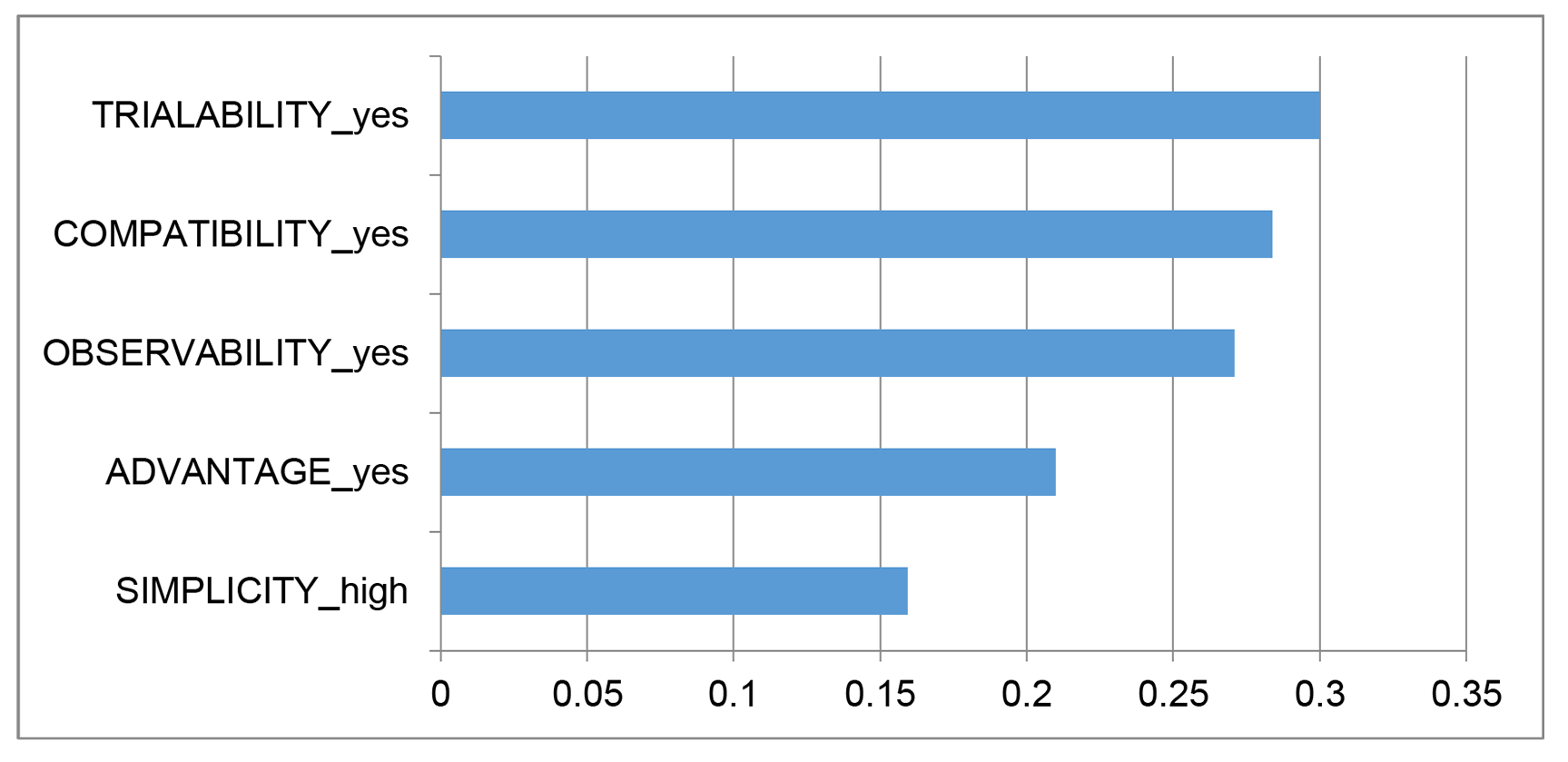

Supplement: S1 Fig — (TIF) [file pone.0202014.s005.tif]
